# MeCas12a, a Highly Sensitive and Specific System for COVID‐19 Detection

**DOI:** 10.1002/advs.202001300

**Published:** 2020-09-23

**Authors:** Peixiang Ma, Qingzhou Meng, Baoqing Sun, Bing Zhao, Lu Dang, Mingtian Zhong, Siyuan Liu, Hongtao Xu, Hong Mei, Jia Liu, Tian Chi, Guang Yang, Ming Liu, Xingxu Huang, Xinjie Wang

**Affiliations:** ^1^ Shanghai Institute for Advanced Immunochemical Studies ShanghaiTech University Shanghai 201210 China; ^2^ Affiliated Cancer Hospital & Institute of Guangzhou Medical University 78 Hengzhigang Road Guangzhou 510095 China; ^3^ State Key Laboratory of Respiratory Disease National Clinical Research Center for Respiratory Disease Guangzhou Institute of Respiratory Health The First Affiliated Hospital Guangzhou Medical University Guangzhou 510120 China; ^4^ Microbiological Testing Laboratory Shanghai Pudong New Area Center for Disease Control and Prevention Shanghai 200136 China; ^5^ Institute for Brain Research and Rehabilitation Guangdong Key Laboratory of Mental Health and Cognitive Science Center for Studies of Psychological Application South China Normal University Guangzhou 510631 China; ^6^ School of Life Science and Technology ShanghaiTech University Shanghai 201210 China

**Keywords:** COVID‐19, CRISPR‐based detection, CRISPR/Cas12a, manganese, SARS‐CoV‐2

## Abstract

Cas12a‐based systems, which detect specific nucleic acids via collateral cleavage of reporter DNA, display huge potentials for rapid diagnosis of infectious diseases. Here, the Manganese‐enhanced Cas12a (MeCas12a) system is described, where manganese is used to increase the detection sensitivity up to 13‐fold, enabling the detection of target RNAs as low as five copies. MeCas12a is also highly specific, and is able to distinguish between single nucleotide polymorphisms (SNPs) differing by a single nucleotide. MeCas12a can detect severe acute respiratory syndrome coronavirus 2 (SARS‐CoV‐2) in clinical samples and distinguish between SARS‐CoV‐2 and Middle East respiratory syndrome coronavirus (MERS‐CoV) RNA in simulated samples, thus offering an attractive alternative to other methods for the diagnosis of infectious diseases including COVID‐19 and MERS.

## Graphical abstract

A Manganese‐enhanced Cas12a‐based (MeCas12a) system is developed, enabling the limit of detection to reach five copies and high specificity. The MeCas12a can detect severe acute respiratory syndrome coronavirus 2 (SARS‐CoV‐2) well in clinical samples, and distinguish synthetic SARS‐CoV‐2 and Middle East respiratory syndrome coronavirus (MERS‐CoV) RNAs, thus offering an alternative diagnosis of infectious diseases including COVID‐19 and MERS.

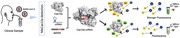

## 1 Introduction

Infectious diseases account for about 22% of all human deaths.^[^ ^1^ ^]^ Rapid detection of pathogenic genetic materials is invaluable for controlling such diseases, especially in resource‐constrained regions.^[^ ^2^ ^]^ Numerous detection assays have emerged, including polymerase chain reaction (PCR)‐based methods, isothermal amplification, and next‐generation sequencing,^[^ ^3^ ^]^ which unfortunately require sophisticated instruments and skills, thus limiting their applications.

Clustered regularly interspaced short palindromic repeats associated (CRISPR/Cas)‐based nucleic acid detection methods have emerged as attractive alternatives to conventional methods. These methods require no special instrument; are simple, sensitive, specific, cheap, and rapid; and can detect multiple pathogens in one assay.^[^ ^4^ ^]^ These new methods exploit the collateral cleavage activity of certain Cas proteins to convert target nucleic acid information into visible signals.^[^ ^5^ ^]^ For example, upon recognition of target single stranded DNA (ssDNA) by Cas12: CRISPR RNA (crRNA) complex, Cas12 becomes activated and randomly cleaves quenched‐florescent ssDNA reporter, releasing the fluorescent dye to yield a visible signal.^[^ ^6^, ^7^, ^8^ ^]^ The same principle underlies Cas13‐based platform.^[^ ^8^ ^]^ CRISPR/Cas systems have been used for detecting various pathogens, including Zika virus, Dengue virus, papillomavirus (HPV), and African swine fever virus (ASFV).^[^ ^7^, ^9^, ^10^, ^11^ ^]^

The devastating COVID‐19 pandemic is caused by SARS‐CoV‐2, with over 7 690 708 confirmed cases and 427 630 related deaths by 14 June 2020.^[^ ^12^ ^]^ Two other coronaviruses, severe acute respiratory syndrome coronavirus (SARS‐CoV) and Middle East respiratory syndrome coronavirus (MERS‐CoV), caused a severe epidemic in 2002 and 2012, respectively.^[^ ^13^ ^]^ The radiology perspective imaging features of COVID‐19 show significant overlap with those of SARS and MERS.^[^ ^14^ ^]^ A recent study on coronavirus showed that recombination might occur between SARS‐CoV‐2 and MERS‐CoV RNA, and this work is in preprint that has not yet been peer reviewed.^[^ ^15^ ^]^ Thus, a diagnostic method for coronaviruses infection and recombined coronaviruses in patient samples should develop. Several Cas12‐ or Cas13‐based methods have been reported for detecting SARS‐CoV‐2, and some of them are reported in preprints that have not yet been peer reviewed.^[^ ^16^ ^]^ Nevertheless, their sensitivities are suboptimal for clinical application.

Divalent cations are capable of promoting pre‐crRNA processing by Cas12a in the activation step,^[^ ^17^ ^]^ or DNA cleavage steps,^[^ ^18^ ^]^ and different divalent ions had distinct contributions to the sensitivity of the Cas13‐based SHERLOCK (specific high‐sensitivity enzymatic reporter unlocking) detection system.^[^ ^9^ ^]^ Therefore, we speculated that the divalent cations might improve the Cas12a detection system. In this work, we screened many divalent ions and found manganese ion (Mn^2+^) effective. The Manganese‐enhanced Cas12a detection (MeCas12a) enhanced the signal up to 13‐folds for the tested crRNAs, and this assay can detect a single digital number of copies of RNA fragments in 45 min without a particular instrument. In addition, the MeCas12a can precisely detect COVID‐19 in clinical samples, and distinguish the mono‐ or co‐infection of SARS‐CoV‐2 and MERS‐CoV in the simulated samples, demonstrating a versatile strategy for COVID‐19 diagnosis.

## 2 Results and Discussion

### 2.1 Design of crRNAs Targeting SARS‐CoV‐2 and MERS‐CoV

For detection of SARS‐CoV‐2 and MERS‐CoV, the *E* gene was selected as the target for Cas12a‐mediated detection as suggested by World Health Organization (WHO).^[^ ^19^ ^]^ Four SARS‐CoV‐2‐specific crRNAs (SC2‐crRNA1–4) and four MERS‐CoV‐specific crRNAs (MC‐crRNA1–4) were designed on the conserved region of each coronavirus strains^[^ ^20^ ^]^ (Figure S1a–c, Supporting Information). The CRISPR/Cas12a‐based detection readout signal relies on the crRNA‐guided targeting cleavage efficiency, which is affected by the spacer sequence and secondary structure of crRNA.^[^ ^21^ ^]^ We then tested the abilities of various crRNAs to guide Cas12 to detect synthetic ssDNA fragments of their respective *E* genes, and found SC2‐crRNA2–4‐ and MC‐crRNA1–3‐induced stronger fluorescence (Figure S2a,b, Supporting Information), which were chosen for subsequent experiments. The potential off‐target was analyzed, and no cross‐reaction was detected (Figures S1d,e and S2c,d, Supporting Information).

### 2.2 Development of the MeCas12a System

**Figure 1:**
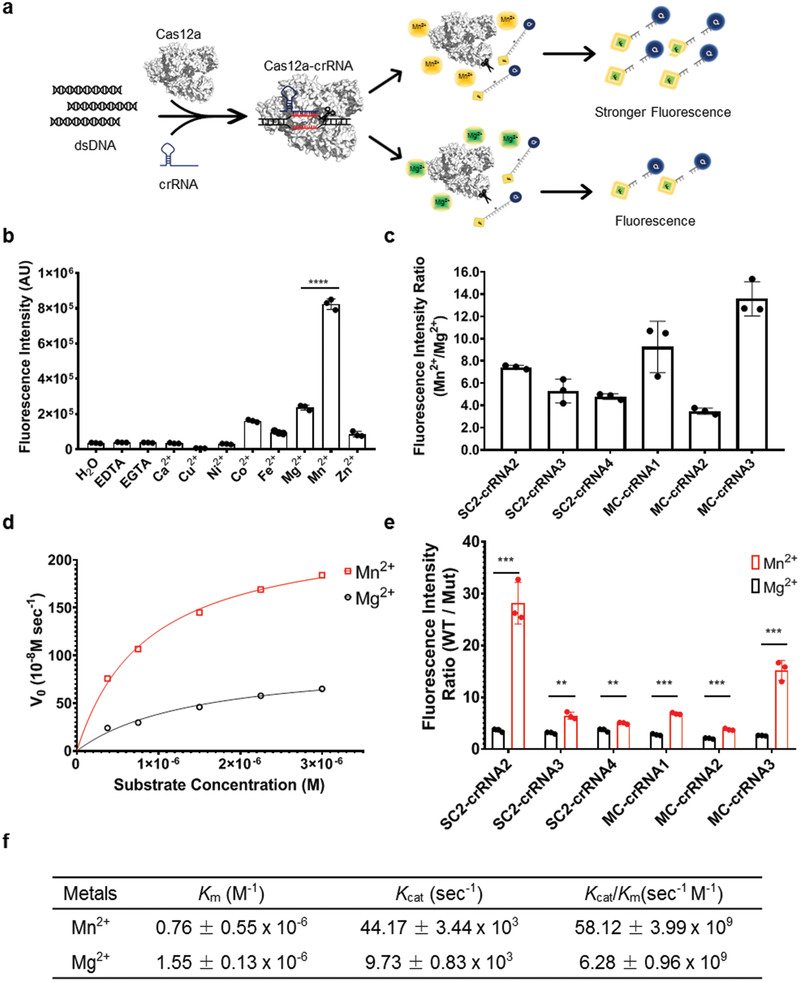
Manganese‐enhanced Cas12a based nucleic acid detection (MeCas12a). a) Schemes of the Manganese‐enhanced Cas12a based biosensing. b) Analysis of divalent cation preference for the Cas12a detection system. c) Enhancement of Cas12a cleavage activity by supplemented with manganese (Mn^2+^) compared to magnesium (Mg^2+^) for different target genes and related crRNAs. d) Michaelis–Menten analysis of the Cas12a cleavage activity with magnesium or manganese. Representative plots of initial velocities versus substrate concentrations in the presence of Mg^2+^ or Mn^2+^, using 0.16 × 10^−9^ m effective Cas12a‐crRNA‐activator complex and increasing substrate concentrations at 37 °C. e) The specificity of Cas12a cleavage of wild type (W.T.) or mutant (Mut) SARS‐CoV‐2 or MERS‐CoV *E* gene in the presence of Mg^2+^ or Mn^2+^. f) Calculated *K*_cat_, *K*_m_, and *K*_cat_/*K*_m_ values were reported as the mean ± standard deviation (s.d.). All the error bars were determined from three independent experiments. Statistical significance between two groups was assessed with an unpaired two‐tailed Student's *t*‐test. Significance was considered as *p < 0.05; **p < 0.01; ***p < 0.001; and ****p < 0.0001.

Next, we screened many divalent cations for their abilities to improve the sensitivity of Cas12a‐mediated nucleic acid detection, including Ca^2+^, Co^2+^, Cu^2+^, Fe^2+^, Mg^2+^, Mn^2+^, Ni^2+^, and Zn^2+^ (**Figure** **1**a). We found that manganese (Mn^2+^) but not Mg^2+^ can markedly increase the readout signal in several crRNA‐induced assays (Figure 1b; Figure S3, Supporting Information). Quantifications indicate that Mn^2+^ produced the signals 3.4–13.6‐fold higher than Mg^2+^ (Figure 1c), with the optimal concentration of Mn^2+^ being around 5 × 10^−3^ m (Figure S4a, Supporting Information). Also, we found that the ssDNA cutting is more efficient in the presence of (100–250) × 10^−3^ m NaCl concentration for Mn^2+^ (Figure S4b, Supporting Information). We also compared the performance of two Cas12 subtypes, AsCas12 and LbCas12a, in the Mn^2+^‐enhanced detection system. As shown in Figure S4c,d (Supporting Information), LbCas12a specifically recognized the target DNA guided by the crRNA and efficiently generated readout signals. In contrast, AsCas12 was nonspecifically activated by the saliva DNA without the target DNA and crRNA in the presence of manganese ions, which is consistent with previous work.^[^ ^22^ ^]^ Therefore, LbCas12a was chosen for further studies.

Cas12a‐catalyzed site‐specific double sranded DNA (dsDNA) cleavage is a single turnover reaction, while the collateral nonspecific ssDNA cleavage is a multiple turnover.^[^ ^7^ ^]^ In the presence of Mn^2+^, the collateral ssDNA cleavage rate is 44 170 turnovers s^−1^ and catalytic efficiency (*k*_cat_/*K*_m_) 58.12 × 10^9^ s^−1^ m ^−1^, much higher than the corresponding values (9730 and 6.28 × 10^9^) in the presence of Mg^2+^ (Figure 1d,f; Figure S5, Supporting Information).

### 2.3 Nucleotide Polymorphism Detection by MeCas12a

We next evaluated the specificity of MeCas12a by introducing one, two, or three mutations in the guide region of the target dsDNA fragment, finding that even a single mismatch can significantly impair MeCas12a biosensing, with the mutations reducing the readout signal 3.7–28× in the presence of Mn^2+^, as compared with only 1.9–3.8× in the presence of Mg^2+^ (Figure 1e; Figures S7 and S8, Supporting Information).

### 2.4 The Detection Limit of MeCas12a

**Figure 2:**
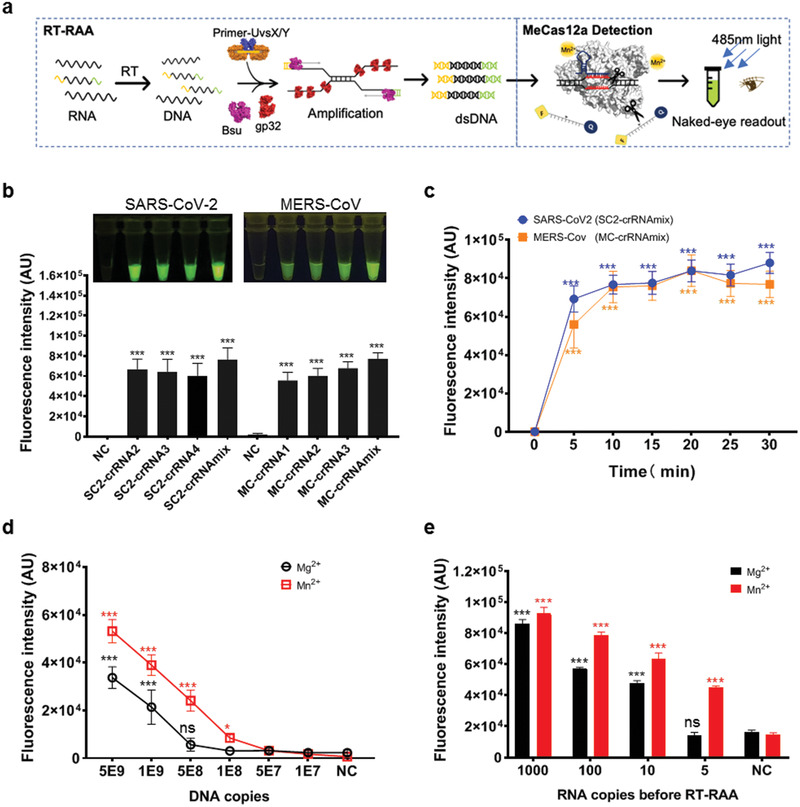
Detection of a single digital copy of target RNA with MeCas12a. a) The workflow of Coronavirus detection using reverse transcript recombinase‐aided amplification (RT‐RAA) coupled MeCas12a detection. b) *E* gene fragments of SARS‐CoV‐2 and MERS‐CoV were recognized by corresponding crRNAs and the crRNAmix. The fluorescent images and intensities at 15 min of the reaction were shown. c) Time‐course detection of SARS‐CoV‐2 and MERS‐CoV *E* genes with SC2‐crRNAmix or MC‐crRNAmix, respectively. d) Gradient diluted SARA‐CoV‐2 *E* gene DNA targets were detected by crRNAmix for SARA‐Cov‐2 in the reaction buffer with Mg^2+^ or Mn^2+^, and the fluorescence intensities at the 15 min point of the reaction were shown. e) Gradient diluted SARA‐CoV‐2 *E* gene RNA fragments were amplified by RT‐RAA, then 10 μL of desalted amplified nucleic acids was detected by crRNAmix of SARA‐CoV‐2 in the reaction buffer with Mg^2+^ or Mn^2+^, and the fluorescence intensity at the 15 min point in the reaction was shown. All the error bars were determined from three independent experiments. Statistical significance for comparisons of more than two groups was determined using one‐way ANOVA. Significance was considered as *p < 0.05; **p < 0.01; ***p < 0.001; and ****p < 0.0001.

To determine the detection limit, we used the MeCas12a system to detect target nucleic acid with or without reverse transcript recombinase aided amplification (RT‐RAA; **Figure** **2**a). We first confirmed that SC2‐crRNA2–4 and MC‐crRNA1–3 could induce visible fluorescence in the presence of the target DNA, and found the crRNA mixtures (SC2‐crRNAmix and MC‐crRNAmix) produced stronger signals compared with single crRNA (Figure 2b). Multiple crRNAs recognizing different regions in the target sequence will activate more Cas12a protein per unit time than single crRNA, thus generating stronger signals (Figure S6, Supporting Information). Therefore, we chose the crRNA mixtures for further detection to maximize the detection signal. Besides, we also ensured the stability of detection by avoiding crRNA recognition deactivation due to single nucleotide polymorphism (SNP) in the target region. According to the time‐course study, the detection time was set at 15 min after MeCas12a reaction (Figure 2c; Figure S9, Supporting Information). Various amounts of *E* gene DNA fragments were added, revealing the detection limit of MeCas12a being 1 × 10^8^ copies of DNA, compared to 5 × 10^8^ copies with Mg^2+^ (Figure 2d; Figure S10a, Supporting Information). In contrast, if RT‐RAA was introduced to preamplify the target coronavirus RNA fragment, MeCas12a could detect as low as five copies of SARS‐CoV‐2 *E* gene RNA fragments, compared to ten copies with Mg^2+^ (Figure 2d; Figure S10b, Supporting Information).

### 2.5 MeCas12a Can Distinguish between SARS‐CoV‐2 and MERS‐CoV

**Figure 3:**
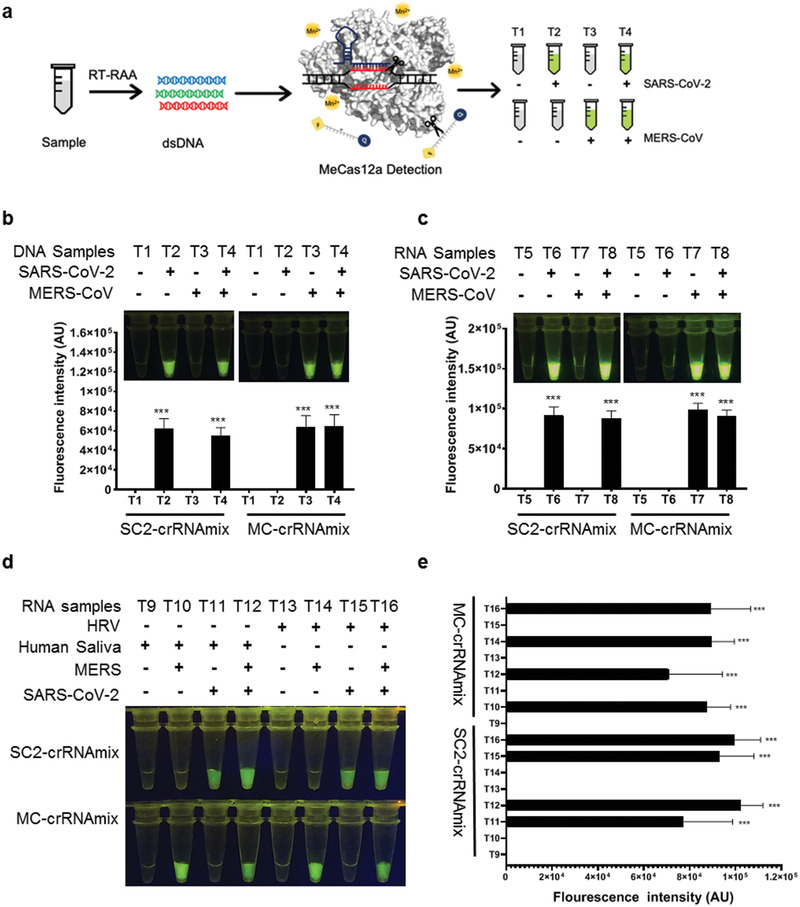
Simulated diagnostic of SARS‐CoV‐2 and MERS‐CoV mono‐ or co‐infection with MeCas12a detection. a) Schemes of the diagnostic workflow of the SARS‐CoV‐2 or MERS‐CoV from stimulated samples. Each sample was detected with SARS‐CoV‐2‐ or MERS‐CoV‐specific crRNAs in parallel. b) Mimicking the diagnostic of mono‐ or co‐infection of SARS‐CoV‐2 and MERS‐CoV with 10^9^ copies of the target *E* gene DNA. The fluorescent signal was read out at the time point after 15 min of the reaction. The fluorescent intensities of data in up were quantified by ImageJ and visualized with GraphPad. c) Simulated diagnostic of SARS‐CoV‐2 or MERS‐CoV co‐infection with synthetic RNA mixed with extracted human saliva RNA. The samples were preamplified using RT‐RAA, then 10 μL of desalted amplified products of each virus were detected by crRNAmix and the fluorescent images at the 15 min of the reaction were shown. The fluorescence intensities of naked‐eye view in up were calculated by software ImageJ and visualized with GraphPad. d) Mimicking detection of SARS‐CoV‐2 or MERS‐CoV with virus RNA. The extract RNA of MERS‐CoV and the transcription of SARS‐CoV‐2 were mixed in ddH_2_O (left) or human rhinovirus (HRV) RNA (right). e) The fluorescent intensities of data in panel (d) were quantified by ImageJ and visualized with GraphPad. All the error bars were determined from three independent experiments. Statistical significance for comparisons of more than two groups was determined using one‐way ANOVA. Significance was considered as *p < 0.05; **p < 0.01; ***p < 0.001; ****p < 0.0001.

The geographic distribution patterns are known to overlap for the confirmed cases of SARS‐CoV‐2 and MERS‐CoV. We, therefore, tested whether MeCas12a can distinguish between the two pathogens. We found that using MeCas12a, SARS‐CoV‐2 crRNA specifically detected the synthetic *E* gene ssDNA fragment from SARS‐CoV‐2 but not that from MERS‐CoV and vice versa, whether the two targets were present alone or together (**Figure** **3**a,b; Figure S11a, Supporting Information). Similar results were obtained when the *E* gene RNA fragments were used as input (Figure 3c; Figure S11b, Supporting Information). To better simulate clinical scenarios, we mixed the SARS‐CoV‐2 or MERS‐CoV RNA template with human saliva RNA or human rhinovirus (HRV) RNA, and found that the impurities did not interfere with MeCas12a's performance (Figure 3d,e).

Furthermore, SARS‐CoV‐2 and MERS‐CoV infections were currently detected in the Kingdom of Saudi Arabia.^[^ ^15^ ^]^ Previous studies demonstrate that coronaviral genomes are prone to recombination.^[^ ^23^ ^]^ A preprint study predicted possible genetic recombination between SARS‐CoV‐2 and MERS‐CoV RNA.^[^ ^15^ ^]^ It is imperative to develop a diagnostic method for recombined coronaviruses. Thus, we designed specific crRNAs that target the potential recombined regions 1 and 3 of SARS‐CoV‐2 and MERS‐CoV (Figure S12a, Supporting Information), and demonstrated that the MeCas12a‐mediated diagnostic can well distinguish between the recombined coronaviruses (Figure S12b–d, Supporting Information).

### 2.6 Performance of MeCas12a on SARS‐CoV‐2 Detection on Clinical Samples

**Figure 4:**
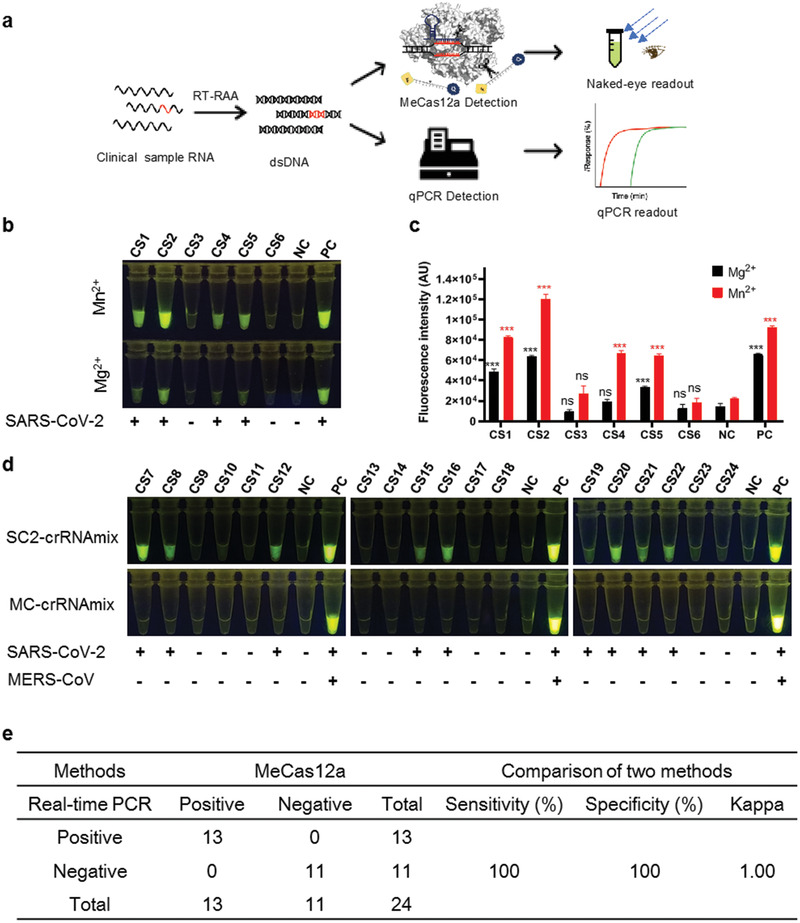
MeCas12a detection of SARS‐CoV‐2 or MERS‐CoV of clinical samples. a) Schematic of coronavirus detection from the clinical samples with both MeCas12a and qPCR. b) Detection of SARA‐CoV‐2 from the clinical sample with Cas12a‐based assay by supplement with Mg^2+^ or Mn^2+^ ions. Sample RNA substrates were amplified by RT‐RAA, then 10 μL of desalted amplified products were detected by SC2‐crRNAmix in the reaction buffer with Mg^2+^ or Mn^2+^, fluorescence intensity at the 15 min point in the reaction were shown. c) The fluorescent intensities of samples were quantified by ImageJ based on data from panel (b). d) The detection of SARS‐CoV‐2 or MERS‐CoV in clinical samples with MeCas12a. Each sample was preamplified with RT‐RAA, then the desalted amplified products were detected with both SC2‐crRNAmix and MC‐crRNAmix for detection of SARS‐CoV‐2 or MERS‐CoV, and the result was determined by the 15 min reaction. The *E* gene RNA and blank served as a positive or negative control in each detection. e) The performance of MeCas12a for SARS‐CoV‐2 detection in clinical samples compared with real‐time PCR. All the error bars were determined from three independent experiments. Statistical significance for comparisons of more than two groups was determined using one‐way ANOVA. Significance was considered as *p < 0.05; **p < 0.01; ***p < 0.001; and ****p < 0.0001.

Finally, we applied MeCas12a to clinical samples (**Figure** **4**a). The RNAs in the samples were amplified and converted into ssRNA with RT‐RAA and then subjected to MeCas12 reaction for 15 min. We first analyzed six clinical samples with MeCas12a in parallel with Mg^2+^ supplemented system. Both methods detected SARS‐CoV‐2 in four of the six samples, but MeCas12a yielded brighter signals, as expected (Figure 4b,c). We then analyzed 18 more clinical samples using MeCas12a, and found 9 of them positive for SARS‐CoV‐2. Besides, all 24 samples were negative for MERS‐CoV (Figure 4d). Quantitative PCR (qPCR) confirmed that 13 of the 24 samples were SARS‐CoV‐2 positive (Table S3, Supporting Information). Thus, MeCas12a and qPCR assays show perfect agreement, with the *κ* value being 1.0 (*p* < 0.001; Figure 4e), confirming the outstanding performance of MeCas12a.

## 3 Conclusion

We have shown that the MeCas12a system, an optimized version of Cas12a detection system, can detect as low as five copies of SARS‐CoV‐2 RNAs. In contrast, Cas12‐ and Cas13‐based detection of SARS‐CoV‐2 requires at least ten copies of viral genome,^[^ ^16^, ^24^ ^]^ similar to PCR‐based methods.^[^ ^3^ ^]^ MeCas12a is thus more sensitive than the other methods. Recently, one study found a significant overlap of the radiology perspective imaging features of COVID‐19 with those of SARS and MERS,^[^ ^14^ ^]^ which may need further nucleic acid detection for the diagnosis of MERS or SARS‐CoV‐2. Importantly, MeCas12a is also highly specific, and is able to distinguish between SARS‐CoV‐2 and MERS‐CoV and between SNPs that differ by a single nucleotide. We also directly demonstrated that MeCas12a could robustly and specifically detect SARS‐CoV‐2 in 24 COVID‐19 patient samples in 45 min.

Given that MeCas12a is cheap, simple to use, and needs no particular instrument or skill, MeCas12a promises to be a superior method for COVID‐19 diagnosis, but more clinical samples need to be tested to verify the results shown in this study. Future work is also required to understand the molecular mechanism behind Mn^2+^‐mediated enhancement.

## 4 Experimental Section

### Clinical Samples and Ethics Statement

Clinical samples of COVID‐19 used in this study were collected and treated in strict accordance with the standard operation of WHO and were approved by the Scientific Research Ethics Review Committee of the First Affiliated Hospital of Guangzhou Medical University. Informed consent was obtained from the patients or from the patients’ legal representatives. Nasopharyngeal swab samples were collected from patients at admission, and RNA was extracted from the samples using the QIAamp RNA Viral Kit (Qiagen, Heiden, Germany). The MERS‐CoV RNA sample was extracted from China GD01 strain (GenBank Accession No. KT006149) from the Guangzhou Institute of Respiratory Health.^[^ ^25^ ^]^ All virus samples were inactivated in a BSL‐3 laboratory, and viral RNA was prepared in a BLS‐2 laboratory.

### Expression and Purification of Recombinant Cas12a Proteins

LbCas12a was expressed and purified as previously described.^[^ ^11^ ^]^ In brief, the coding sequences of LbCas12a were codon‐optimized and synthesized by Genscript (Nanjing, China) and then cloned into pET28a (Novagen) with a C‐terminal 10× His tag. The coding sequence of AsCas12 was synthesized by Genscript (Nanjing, China) as previously described.^[^ ^26^ ^]^ The pET28a–Cas12a plasmid was transformed into *Escherichia coli* BL21 (DE3) and induced with 0.2 × 10^−3^ m Isopropyl ß‐D‐1‐thiogalactopyranoside （IPTG) for 16 h at 18 °C before the cell harvesting. After cell pellets lysis, the Cas12a protein was purified using a Ni–NTA (Nickel‐nitrilotracetic acid) resin column and heparin sepharose column according to the manufacturer's instructions (G.E. Healthcare). Then the purified Cas12a protein was concentrated into storage buffer (50 × 10^−3^ m Tris‐HCl, pH 7.5, 500 × 10^−3^ m NaCl, 10% (v/v) glycerol, 2 × 10^−3^ m dithiothreitol), quantitated using the BCA Protein Assay Kit (Thermo Fisher Scientific, MA, USA), and frozen at −80 °C until use.

### Nucleic acid Preparation

The *E* gene fragment of SARS‐CoV‐2 (Wuhan‐1 strain, GenBank: MN908947), *E* gene fragment form MERS‐CoV (England‐1 strain, GeneBank: KC164505) were synthesized by GenScript (Nanjing, China) and cloned into the pUC57 vector with a T7 primer. The crRNAs were synthesized by GenScript (Nanjing, China), and sequences are listed in Table S1 (Supporting Information).

The RNA fragments of the target gene were cloned with thein vitro transcription (IVT) primers and transcribed using the MEGAshortscript T7 Transcription Kit (Thermo Fisher Scientific, MA, USA). Then RNAs were purified with the MEGAclear Kit (Thermo Fisher Scientific, MA, USA) and recovered by alcohol precipitation according to the manufacturer's instructions. RNAs were aliquot and stored at −80 °C till used. The primers used for in vitro transcription are listed in Table S2 (Supporting Information).

To address the potential off‐target issue and cross reaction with the human genome, three human cell lines (HEK293, Hela, and A549 cell) were used as the source of human genome. HEK293 and Hela cells were maintained in high‐glucose Dulbecco's modified Eagle's medium (DMEM; Hyclone, SH30243), supplemented with 10% heat‐inactivated fetal bovine serum (FBS; Gibco, 10099141). A549 cells were maintained in F‐12K medium (Hyclone, SH30526) and supplemented with 10% heat‐inactivated FBS. The cells were cultured at 37 °C in an incubator with 5% CO_2_. Total cell RNA was extracted from the cultured cells using total RNA extraction reagent (Vazyme, R401‐01), following the manufacturer's instructions. Briefly, 1 × 10^7^ cells were lysed in 1 mL RNA extraction agent. Total RNA was extracted by chloroform and mixing thoroughly. After centrifuging at 12 000 × *g* for 15 min, the supernatant was mixed with an equal volume of isopropanol and centrifuged at 12 000 × *g* for 10 min. After washing with 75% ethanol, the RNA pellet was dissolved in RNase‐free water and stored at −80 °C.

Saliva RNA was extracted from the saliva sample using QIAzol Lysis Reagent (QIAGEN, 79306) following the manufacturer's instructions. Briefly, 1 mL of saliva sample was centrifuged at 13 000 × *g* for 15 min at 4 °C, and the pellet was lysed by 1 mL of QIAzol. Saliva RNA was extracted by chloroform for two times. The chloroform was mixed with chilled isopropanol and centrifuged at 13 000 × *g* for 20 min. After washing with 75% ethanol, the RNA pellet was dissolved in RNase‐free water and stored at −80 °C. Saliva DNA was extracted from the saliva sample using saliva gDNA Isolation Kit (Thermo Fisher, A39059) following the manufacturer's instructions. In brief, the saliva sample was mixed with DNA Lysis/Binding Bead. Next, shaking at 800 rpm for 5 min, the supernatant was discarded. After washing three times, the DNA was eluted in the elution buffer.

### Cas12a‐Mediated Nucleic Acid Detection

The detection assays were performed as previously reported with minor modifications.^[^ ^11^ ^]^ In a 20 µL detection assay, with 200 ng LbCas12a protein, 25 × 10^−12^ m ssDNA FQ probe sensor, 1 × 10^−6^ m crRNA, and 2 µL of the sample in a reaction buffer (100 × 10^−3^ m NaCl, 50 × 10^−3^ m Tris‐HCl, 100 µg mL^−1^ BSA(bovine serum albumin), pH 7.9) supplied with 10 × 10^−3^ m MgCl_2_ or MnSO_4_, were incubated at 37 °C until detection. A PerkinElmer EnSpire reader with the excitation at 485 nm and emission at 520 nm was used for fluorescence detection. The naked‐eye determination was taken under 485 nm light and photographed with a mobile phone. For the divalent‐ion preference screen, the detection assay was supplemented with 10 × 10^−3^ m EDTA (Ethylenediaminetetraacetic acid), EGTA (Ethylene glycol tetraacetic acid), CaCl_2_, CoCl_2_, CuSO_4_, FeSO_4_, NiSO_4_, MgSO_4_, MnSO_4_, or ZnSO_4_.

For Michaelis–Menten analysis, Cas12a–crRNA–activator (target dsDNA) complexes were prepared as described above, and reaction was initiated by diluting Cas12a complexes to a final concentration of 32 × 10^−9^ m LbCas12a:50 × 10^−9^ m SC2‐crRNA:0.16 × 10^−9^ m DNA activator (effective complex = 0.16 × 10^−9^ m, SARS‐CoV‐2 *E* gene) in a solution containing 1× reaction buffer and 0.38 × 10^−6^, 0.75 × 10^−6^, 1.5 × 10^−6^, 2.3 × 10^−6^, and 3 × 10^−6^ m of fluorescence‐quenched ssDNA substrate (GenScript, China). Reactions were incubated in a fluorescence plate reader for up to 30 min at 37 °C with fluorescence measurements being taken at every 60 s (*λ*_ex_: 485 nm; *λ*_em_: 520 nm). The initial velocity (*V*_0_) was calculated by fitting to linear regression and plotted against the substrate concentration to determine the Michaelis–Menten constants (GraphPad Software), according to the following equation: *Y* = (*V*_max_ × *X*)/(*K*_m_ + *X*), where *X* is the substrate concentration and *Y* is the enzyme velocity. The turnover number (*k*_cat_) was determined by the following equation: *k*_cat_ = *V*_max_/*E*_t_, where *E*_t_ = 0.16 × 10^−9^ m.

### Isothermal Amplification Coupled MeCas12a Detection

The RAA or RT‐RAA was introduced for the target gene preamplification by using a commercial RAA kit (Qitian Biological Co., Ltd., Jiangsu, China) according to the manufacturer's instructions. Briefly, in a 50 µL reaction assembled with 25 µL of reaction buffer, 2 µL of forward primer (10 × 10^−6^ m), 2 µL of reverse primer (10 × 10^−6^ m), *x* µL of the sample, 18.5 − *x* of RNase‐free double distilled H_2_O (ddH_2_O), and 2.5 µL of magnesium acetate (280 × 10^−3^ m) were mixed gently before incubating at 39 °C for 30 min. Then, the RAA or RT‐RAA products were desalted using the Zeba column (Cat. 89882, Thermo Fisher Scientific). The desalted sample was transferred to the Cas12a‐mediated detection assay system.

### qPCR Assay

The TaqMan “real‐time PCR” detection of the SARS‐CoV‐2 *E* gene was carried out using a Quant Studio 5 system (Applied Biosystems, MA, USA) according to the WHO recommended procedure (https://www.cdc.gov/coronavirus/2019-ncov/lab/rt-pcr-detection-instructions.html) and detected with commercial TaqMan assay kit (Q222‐cn, Vazyme Biotech Co., Ltd., Nanjing, China) according to the manufacturer's instructions. The amplification primers and probes are shown in Table S2 (Supporting Information). Under the conditions of all positive and negative controls exhibiting the expected performance, the specimen of which amplification plots rise distinctly within the 38.9 cycles (*C*_t_ < 40) could be judged to be positive.

### Statistical Analysis

All experimental results were shown as mean ± standard error of the mean (SEM) unless stated otherwise. One‐way analysis of variance (ANOVA) was used when comparing more than two groups. When only two groups were compared, statistical significance was assessed with an unpaired Student's *t*‐test. Significance was considered as *p < 0.05; **p < 0.01; ***p < 0.001; and ****p < 0.0001. Statistical analyses were carried out with GraphPad Prism 8.0.

## Conflict of Interest

The authors declare no conflict of interest.

## Author Contributions

P.M., Q.M., B.S., and B.Z. contributed equally to this work. P.M., M.L., X.H., and X.W. conceived the study. P.M., X.W., X.H., M.L., J.L., T.C., and G.Y. designed the experiments. P.M., Q.M., and X.W. performed the experiments with the assistance of M.Z., L.D., S.L., H.M., H.X., Y.L., B.Z., and M.L. collected and processed materials. P.M., T.C., M.L., X.H., and X.W. wrote the manuscript.

## Supporting information

Supporting Information
