# Supplementary material for: MeCas12a, a Highly Sensitive and Specific System for COVID‐19 Detection
Source: Adv Sci (Weinh). 2020 Sep 23;7(20):2001300. doi: 10.1002/advs.202001300 (PMC7536916; doi:10.1002/advs.202001300)
Supplement: Supplementary file 1 — Supporting Information [file ADVS-7-2001300-s001.pdf]

Copyright WILEY-VCH Verlag GmbH & Co. KGaA, 69469 Weinheim, Germany, 2020.

Supporting Information

MeCas12a, a highly sensitive and specific system for COVID-19 detection

Peixiang Ma, Qingzhou Meng, Baoqing Sun, Bing Zhao, Lu Dang, Mingtian Zhong, Siyuan Liu, Hongtao Xu, Hong Mei, Jia Liu, Tian Chi, Guang Yang, Ming Liu, Xingxu Huang *, Xinjie Wang**

Supplementary Figures and Tables:

Contents

Figure S1 2
Figure S2 3
Figure S3 4
Figure S4 6
Figure S5 7
Figure S6 8
Figure S7 9
Figure S8 10
Figure S9 11
Figure S10 13
Figure S11 15
Figure S12 17
Table S1 18
Table S2 19
Table S3 20

Figure S1

a

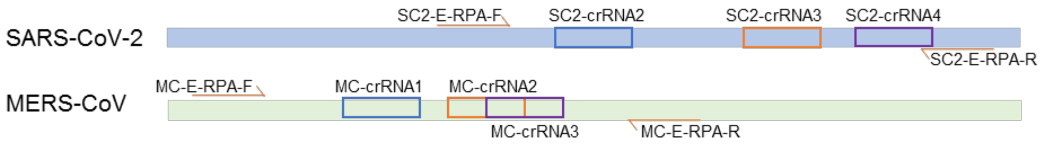

b

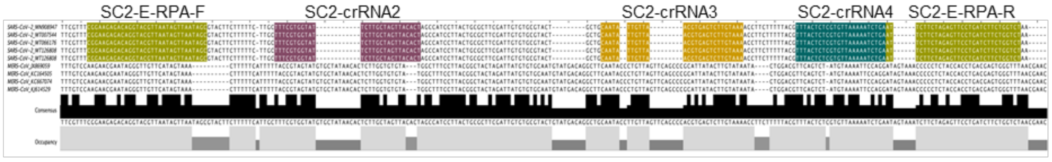

c

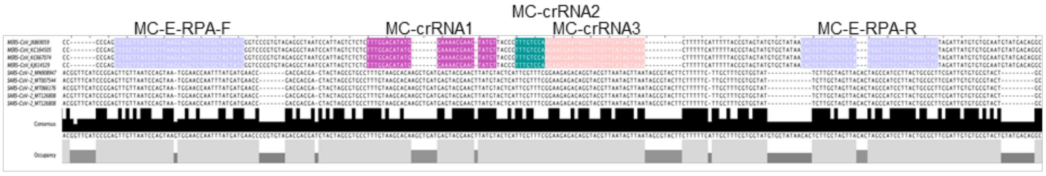

d

crRNA name	crRNA target sequence	Mismatch (SNPs)	Mismatche DNA sequence	Chromosome	Position	Direction
SC2-crRNA1	TTTVTTGCTTTCGTGGTATTCTTGCTA	Yes (3)	TTTGTTGCTTTCtTGGcATTtTTGCTA	chr10	122629542	+
SC2-crRNA2	TTTVGTGGTATTCTTGCTAGTTACACT	No	-	-	-	
SC2-crRNA3	TTTVCAAGACTCACGTTAACAATATTG	No	-	-	-	
SC2-crRNA4	TTTVCTCTCGTGTTAAAAATCTGAATT	No	-	-	-	
MC-crRNA1	TTTVGACATATGGAAAACGAACTATGT	No	-	-	-	
MC-crRNA2	TTTVTCCAAGAACGAATAGGGTTGTTC	No	-	-	-	
MC-crRNA3	TTTVCTATGAACAACCCTATTCGTTCT	No	-	-	-	
MC-crRNA4	TTTVCATATGTCCAAAGAGAGACTAAT	Yes (3)	TTTCCtTAaGTCCAAAGAGAGACTAAg	chr2	173749475	+

e

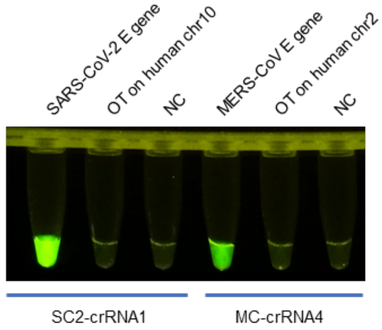

Figure S1. Design of specific crRNAs for SARS-CoV-2 or MERS-CoV detection targeting at E gene. (**a**) Sequence alignment of SC2-crRNAs for SARS-CoV-2 (**b**) and MC-crRNAs for MERS-CoV (**c**) with primers for RT-RAA. (**d**) Potential off-target sites on the human genome. (**e**) The potential off-target genes were cloned and validated for SC2-crRNA1 and MC-crRNA4.

Figure S2

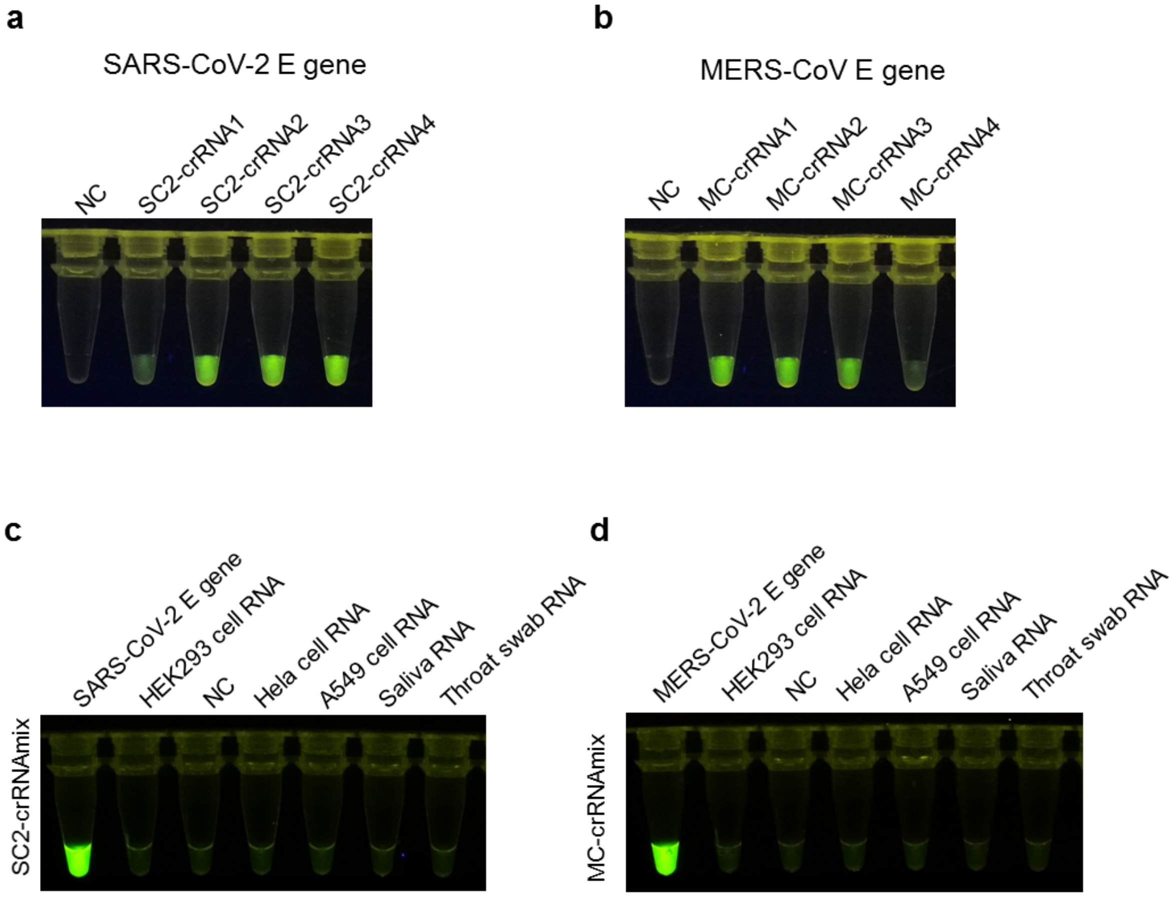

Figure S2. Validation of the theoretically designed crRNAs targeting at E gene fragments of SARS-CoV-2 (**a**) or MERS-CoV (**b**). The fluorescence intensities were used to evaluate the efficiency, and the top 3 efficient crRNAs were used in further experiments. Detection of potential off-target effects using human RNA. The RNA was amplified with reverse transcription RPA (RT-RPA) and detected by MeCas12a guided by SC2-crRNAmix (**c**) or MC-crRNAmix (**d**). The human RNA were extrated from HEK293 cell line, Hela cell line, A549 cell line, human saliva or throat swab samples.

Figure S3

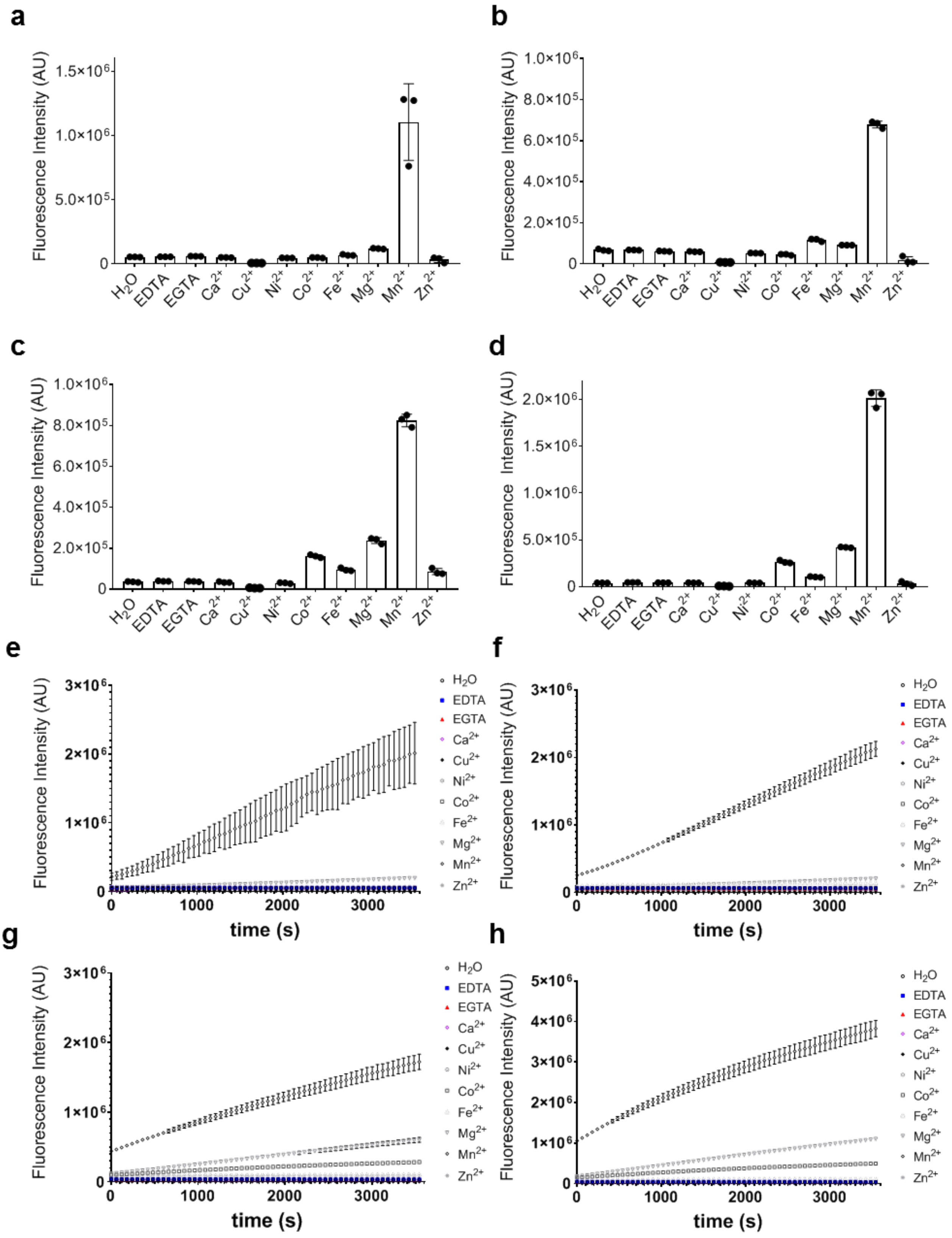
a
b
c
d
e
f
g
h
Fluorescence Intensity (AU)
H2O
EDTA
EGTA
Ca2+
Cu2+
Ni2+
Co2+
Fe2+
Mg2+
Mn2+
Zn2+
time (s)

Figure S3. Divalent ion preference of Cas12a for fluorescent-quenched ssDNA cleavage. MERS-CoV E gene and related MC-crRNA-2 (**a, e**) and MC-crRNA-3 (**b, f**) and SARS-CoV-2 E gene and related SC2-crRNA-2 (**c, g**) and SC2-crRNA-3 (**d, h**) were incubated with different divalent ions. EGTA, EDTA and H_2O were used as internal controls. The fluorescence intensities at the 15-minute (**a, b, c, d**) or time-course analysis for 1 hour (**e, f, g, h**) were shown.

Figure S4

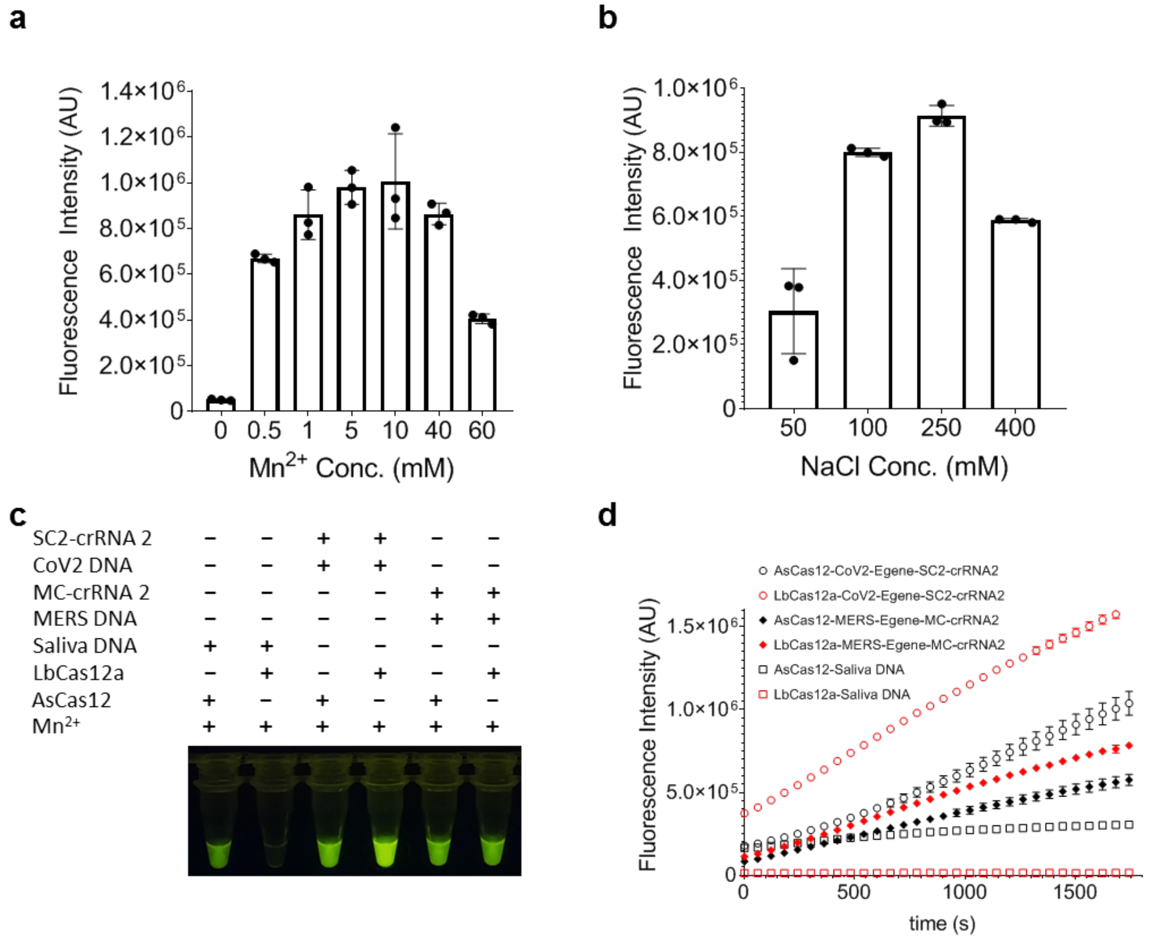

Figure S4. Optimization of MeCas12a detection. **(a)** Series concentrations of Manganese ions were added to the Cas12a based detection system. The fluorescence signals from cleaved fluorescent dye were used to evaluate the cleavage efficiency. (**b)** Series concentrations of NaCl were supplemented to the MeCas12a reaction. The fluorescence signals from cleaved fluorescent dye were used to evaluate the cleavage efficiency. The error bars were determined from 3 independent experiments. The activity of AsCas12 and LbCas12 for fluorescent-quenched ssDNA cleavage **(c, d)**. SARS-CoV-2 *E* gene and related SC2-crRNA-2, MERS-CoV *E* gene and related MC-crRNA-2, and saliva DNA were used to analyze the activity of AsCas12 and LbCas12a, respectively. The fluorescent images at the 15-min (**c**) or time-course analysis for 30 min (**d**) were shown.

Figure S5

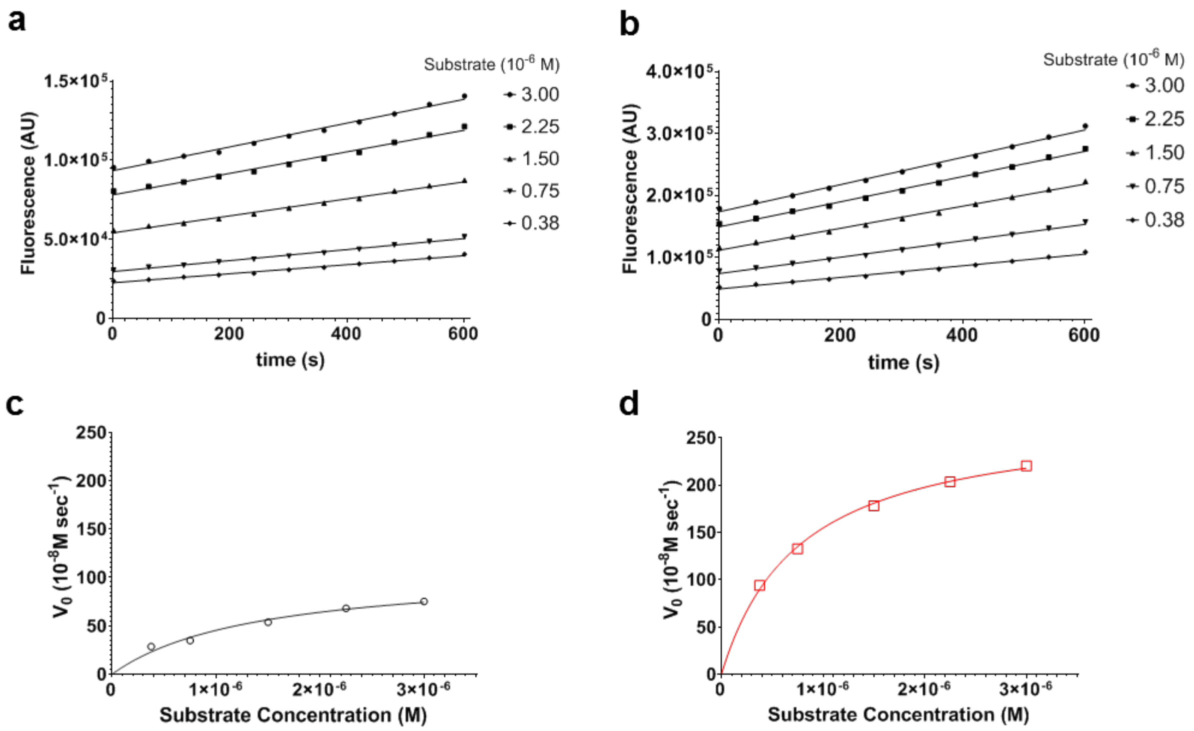

Figure S5. Michaelis-Menten analysis of the Cas12a cleavage activity with Magnesium or Manganese. Representative plots of initial velocity versus time in presence of (**a**) Mg^{2+} or (**b**) Mn^{2+}, using 0.16 nM effective LbCas12a-crRNA-activator complex and increasing substrate concentrations at 37°C. Michaelis-Menten fits for the corresponding (**c**) Mg^{2+} or (**d**) Mn^{2+}.

Figure S6

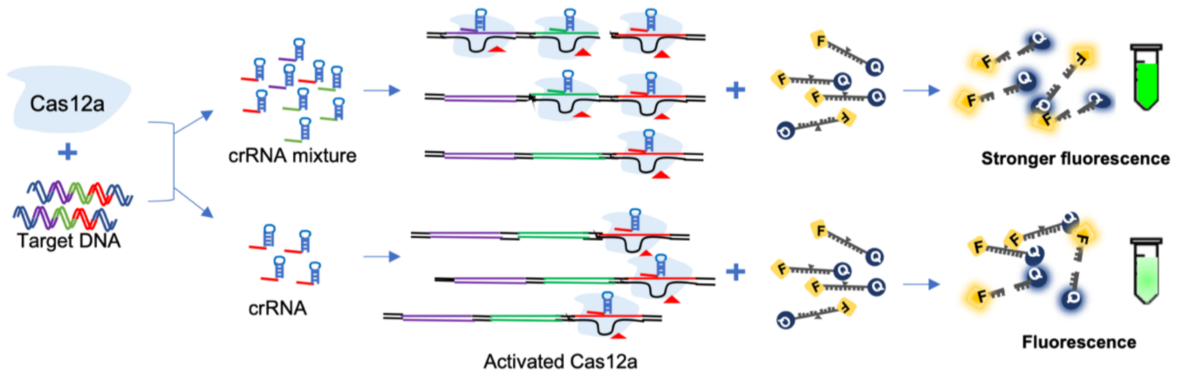

Figure S6. Schematic diagram showing that multiple crRNAs mediate stronger readout signal. In the detection system, there is an equal amount of individual crRNA, Cas12a protein, fluorescence reporter and input DNA.

Figure S7

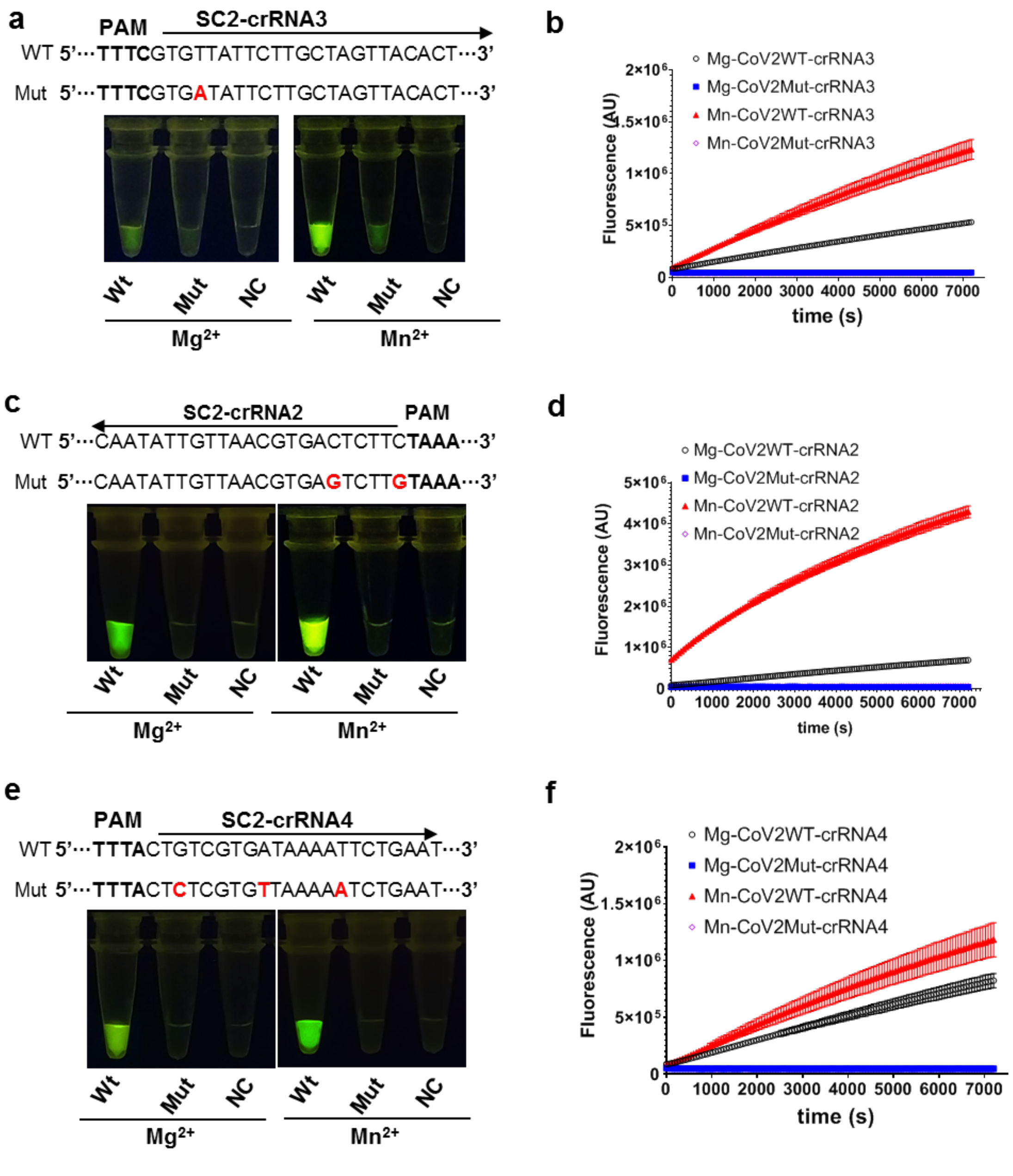

Figure S7. The specificity of different target genes and crRNAs in the presence of Magnesium or Manganese divalent ions. Detection of wild type (WT) *E* gene DNA fragments of the SARS-CoV-2 compared with one (**a**), two (**b**) or three SNPs (Mut) (**c**) which were recognized by the corresponding SC2-crRNAs in the buffer with Mg^{2+} or Mn^{2+}. The fluorescent images at the 15-minute (**a, c, e**) or 2 h (**b, d, f**) time-course fluorescence were shown to compare the specificity in the presence of Mg^{2+} or Mn^{2+}.

Figure S8

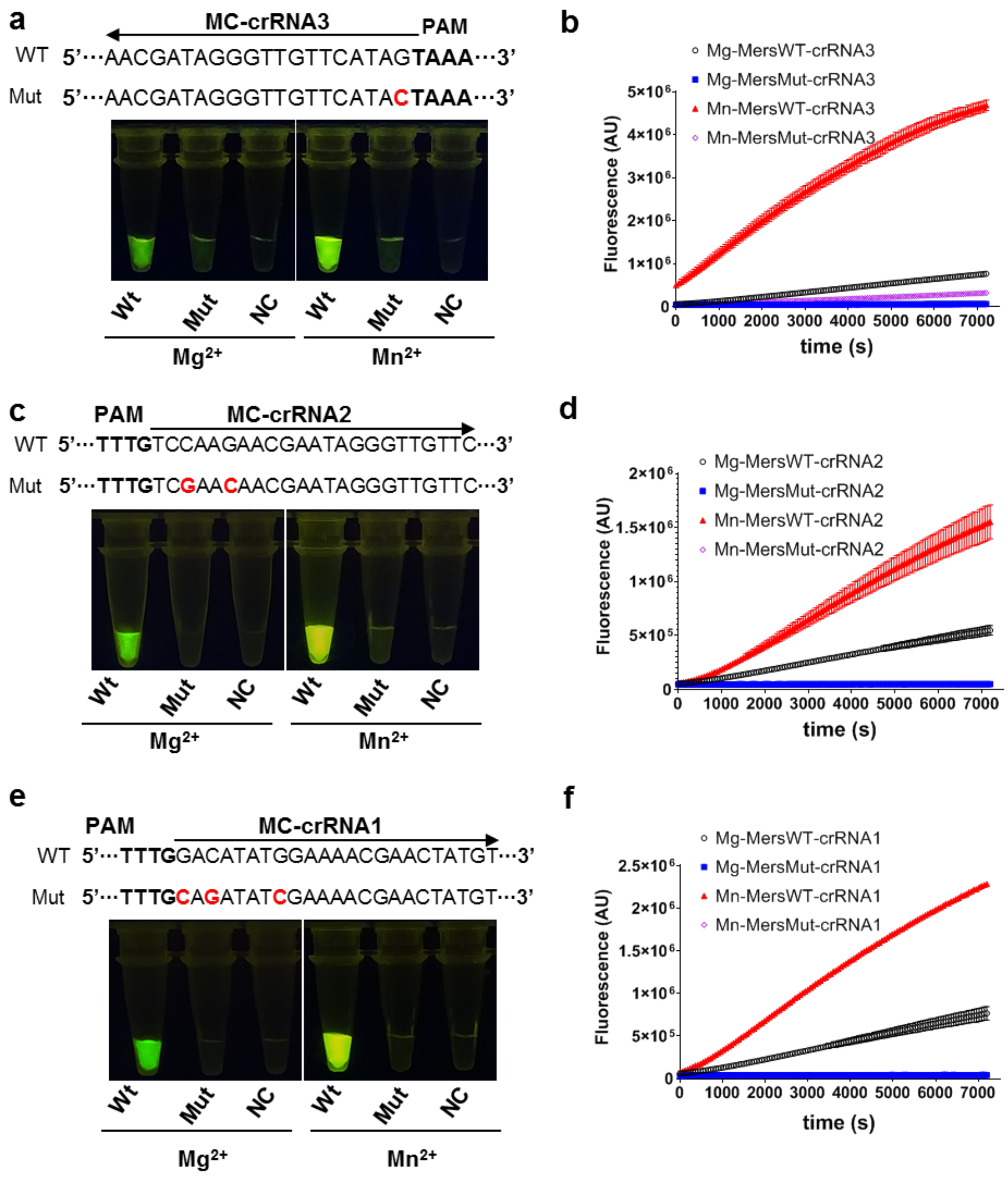

Figure S8. The specificity of different target genes and crRNAs in the presence of Magnesium or Manganese divalent ions. Detection of wild type (WT) *E* gene DNA fragments of the MERS-CoV compared with one (**a**), two (**b**) or three SNPs (Mut) (**c**) which were recognized by the corresponding MC-crRNAs in the buffer with Mg^{2+} or Mn^{2+}. The fluorescent images at the 15-minute (**a, c, e**) or 2 h (**b, d, f**) time-course fluorescence were shown to compare the specificity in the presence of Mg^{2+} or Mn^{2+}.

Figure S9

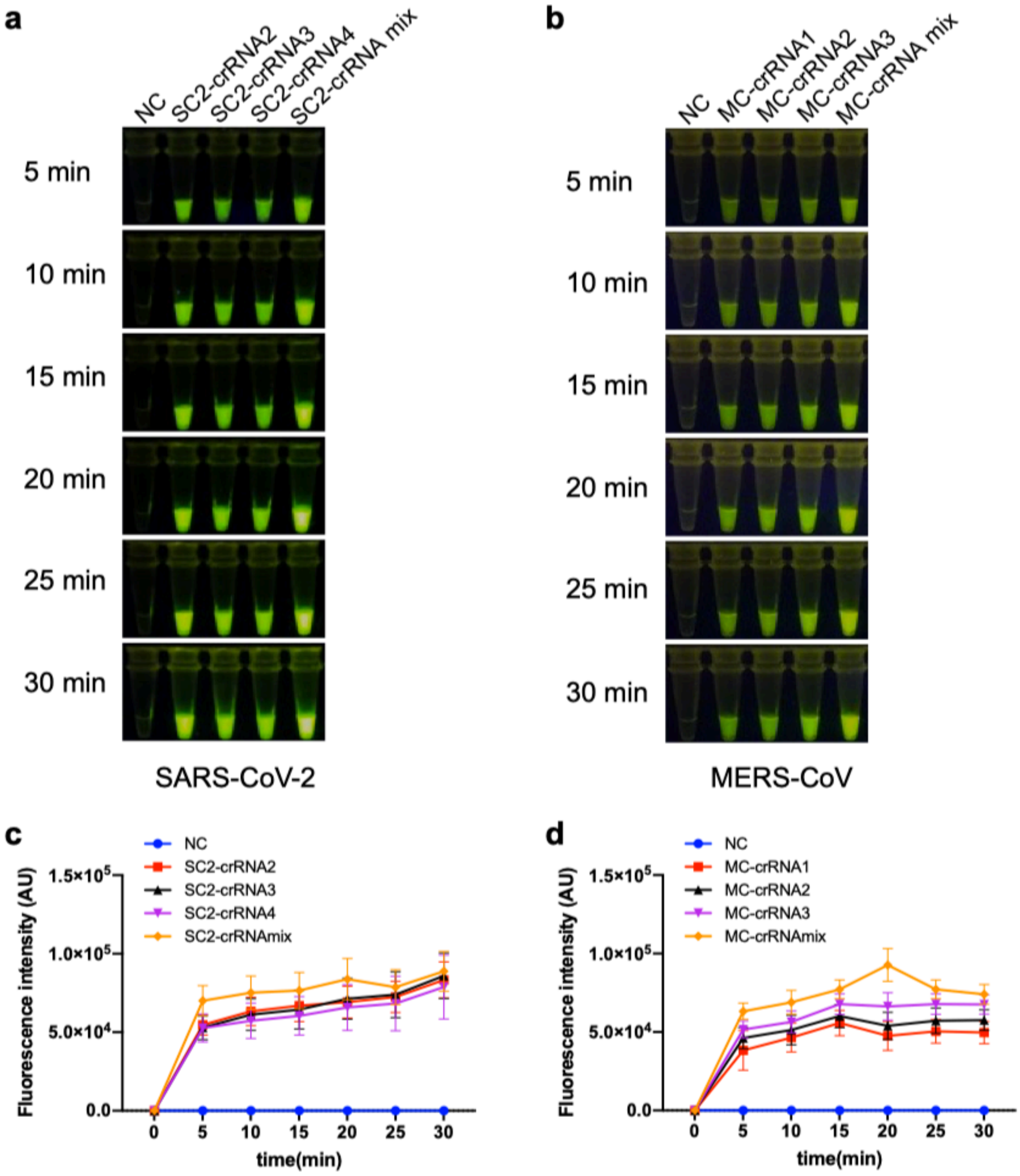

Figure S9. The reaction of cRNA or the crRNAmix with the SARS-CoV-2 (**a**) and MERS-CoV (**b**) were detected with their respective targeted DNA substrate and the fluorescent images at different time points were shown. The time-course analysis of fluorescence intensity of LbCas12a preassembled with a crRNA targeting SARS-CoV-2 (**c**) or MERS-CoV (**d**) in the presence of target DNA fragment and ssDNA FQ probe. The intensity of fluorescence (per tube) was quantified according to the fluorescent pictures taken every 5 min for 30 min at 37°C.

The data was shown as the mean ± s.d. All the error bars were determined from 3 independent experiments.

Figure S10

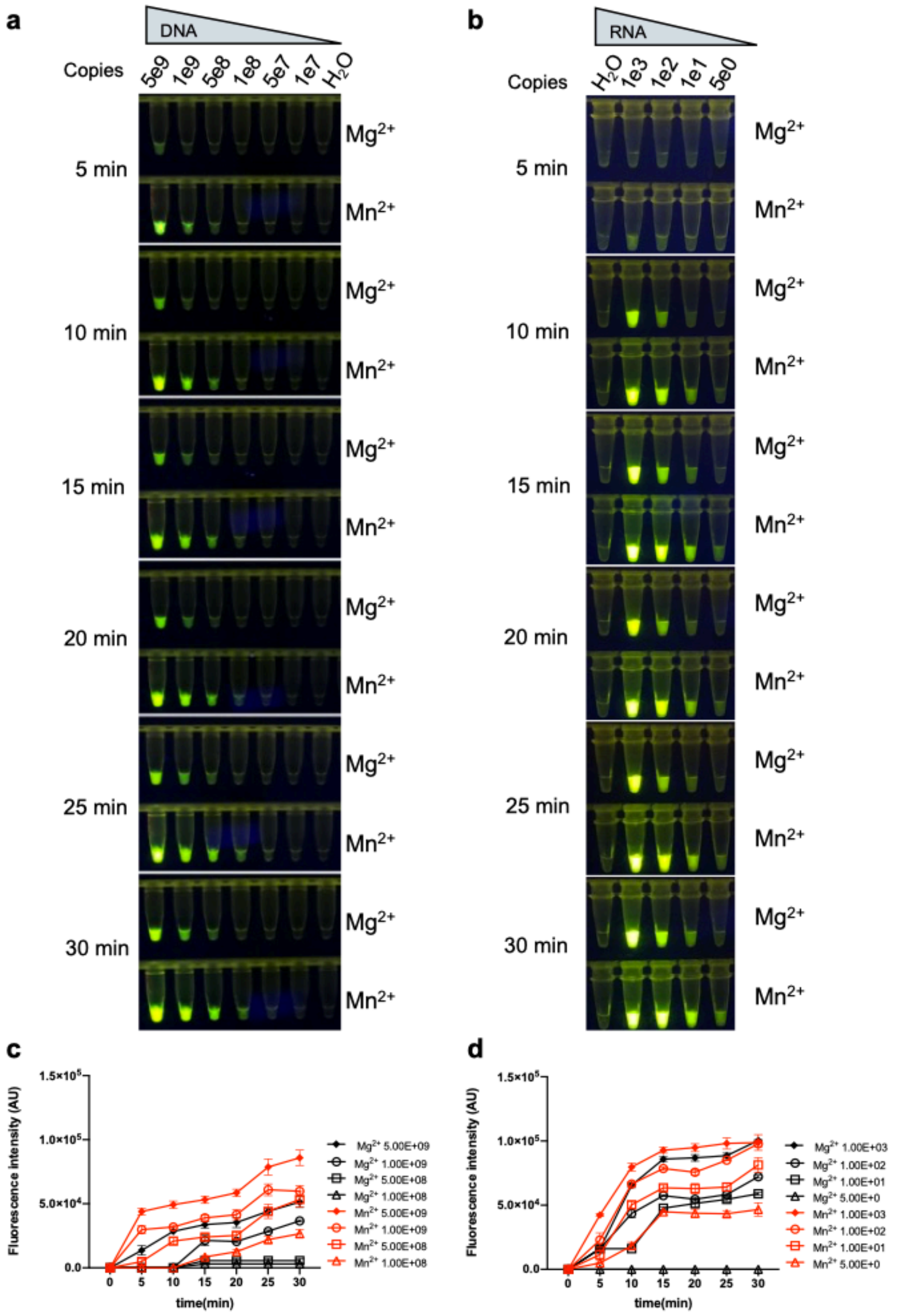

Figure S10. The detection limit of Cas12a mediated assay in the reaction buffer supplemented with Mg^{2+} or Mn^{2+} ions. (**a**) Gradient diluted SARA-Cov-2 *E* gene DNA substrate was detected by crRNAmix of SARA-Cov-2 in the reaction buffer supplemented with Mg^{2+} or Mn^{2+} ions, fluorescent images and fluorescence intensities were recorded every 5 minutes. (**b**) Gradient diluted SARA-CoV-2 *E* gene RNA substrates were amplified by RT-RAA, then 10 microliters of desalted substrate were detected by crRNAmix of SARA-Cov-2 in the reaction buffer with

Mg^{2+} or Mn^{2+}, fluorescent images at different time points in the reaction were shown. The fluorescence intensity (per tube) for SARS-CoV-2 *E* gene DNA (**c**) and RNA (**d**) were quantified according to the fluorescence pictures taken every 5 min for 30 min at 37°C. The data was shown as the mean ± s.d. All the error bars were determined from 3 independent experiments.

Figure S11

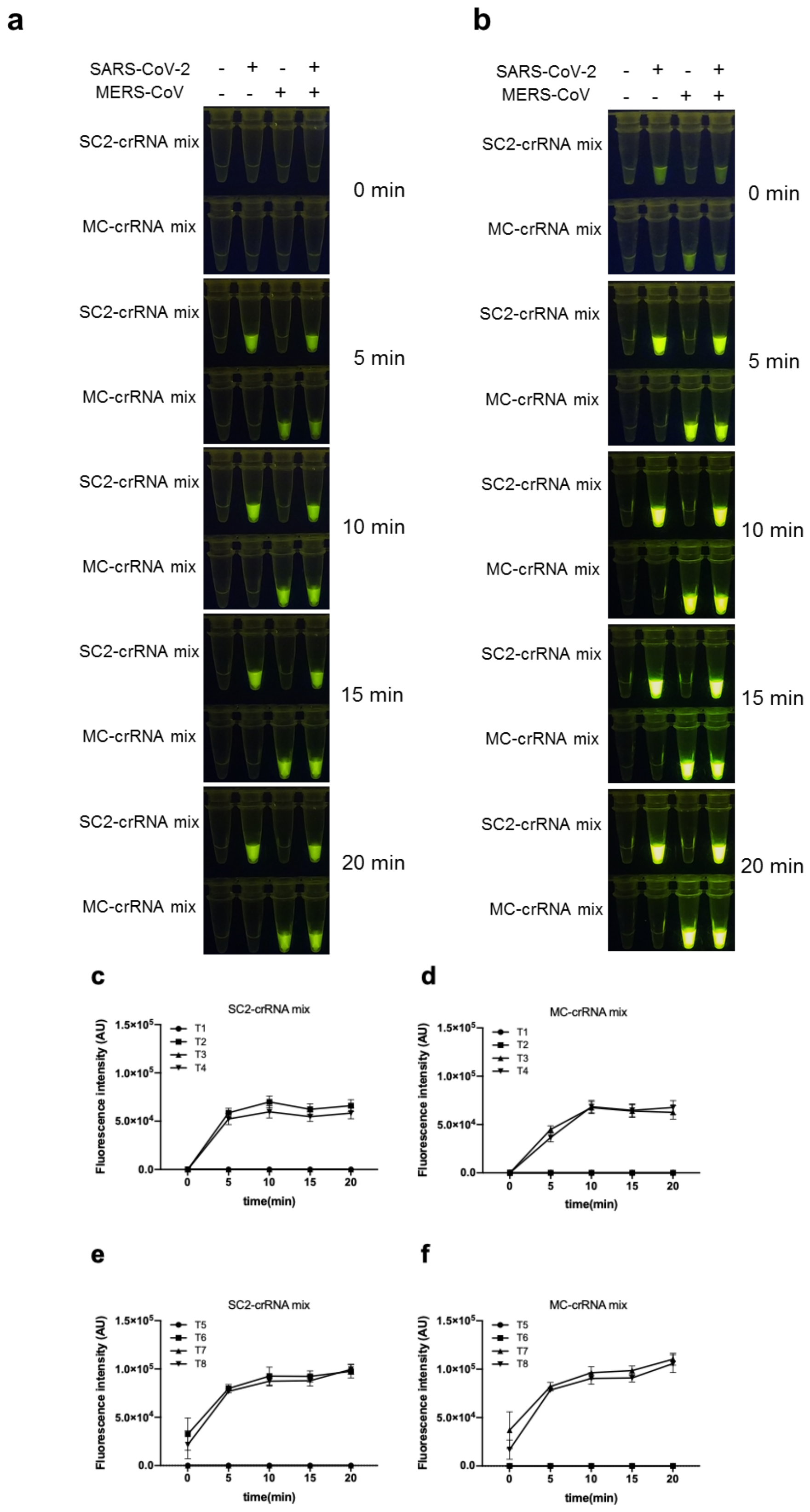
a
SARS-CoV-2 - + - +
MERS-CoV - - + +
SC2-crRNA mix
MC-crRNA mix
0 min
5 min
10 min
15 min
20 min
b
SARS-CoV-2 - + - +
MERS-CoV - - + +
SC2-crRNA mix
MC-crRNA mix
0 min
5 min
10 min
15 min
20 min
c
SC2-crRNA mix
Fluorescence intensity (AU)
time(min)
T1
T2
T3
T4
d
MC-crRNA mix
Fluorescence intensity (AU)
time(min)
T1
T2
T3
T4
e
SC2-crRNA mix
Fluorescence intensity (AU)
time(min)
T5
T6
T7
T8
f
MC-crRNA mix
Fluorescence intensity (AU)
time(min)
T5
T6
T7
T8

Figure S11. Time-course of mimic diagnostic of SARS-CoV-2 and MERS-CoV co-infection. (**a**) DNA substrate of SARS-CoV-2 and MERS-CoV *E* gene were detected by the SC2-crRNAmix and MC-crRNAmix, and the fluorescent images were recorded every 5 minutes. (**b**) Stimulate diagnostic of SARS-CoV-2 and MERS-CoV with the *E* gene RNA substrates. The sample was amplified by RT-RAA then 10 microliters of desalted substrate were detected by SC2-crRNAmix and MC-crRNAmix and the fluorescent images at the different time points of the reaction were shown. The time-course analysis of fluorescence of T1, T2, T3 and T4 samples detected by LbCas12a preassembled with crRNA mixture targeting SARS-CoV-2 (**c**) or MERS (**d**). The time-course analysis of fluorescence of T5, T6, T7 and T8 samples detected by LbCas12a preassembled with crRNA mixture targeting SARS-CoV-2 (**e**) or MERS (**f**). The fluorescence intensity (per tube) was quantified according to the fluorescence pictures taken every 5 min for 30 min at 37°C. The data was shown as the mean ± s.d. All the error bars were determined from 3 independent experiments.

Figure S12

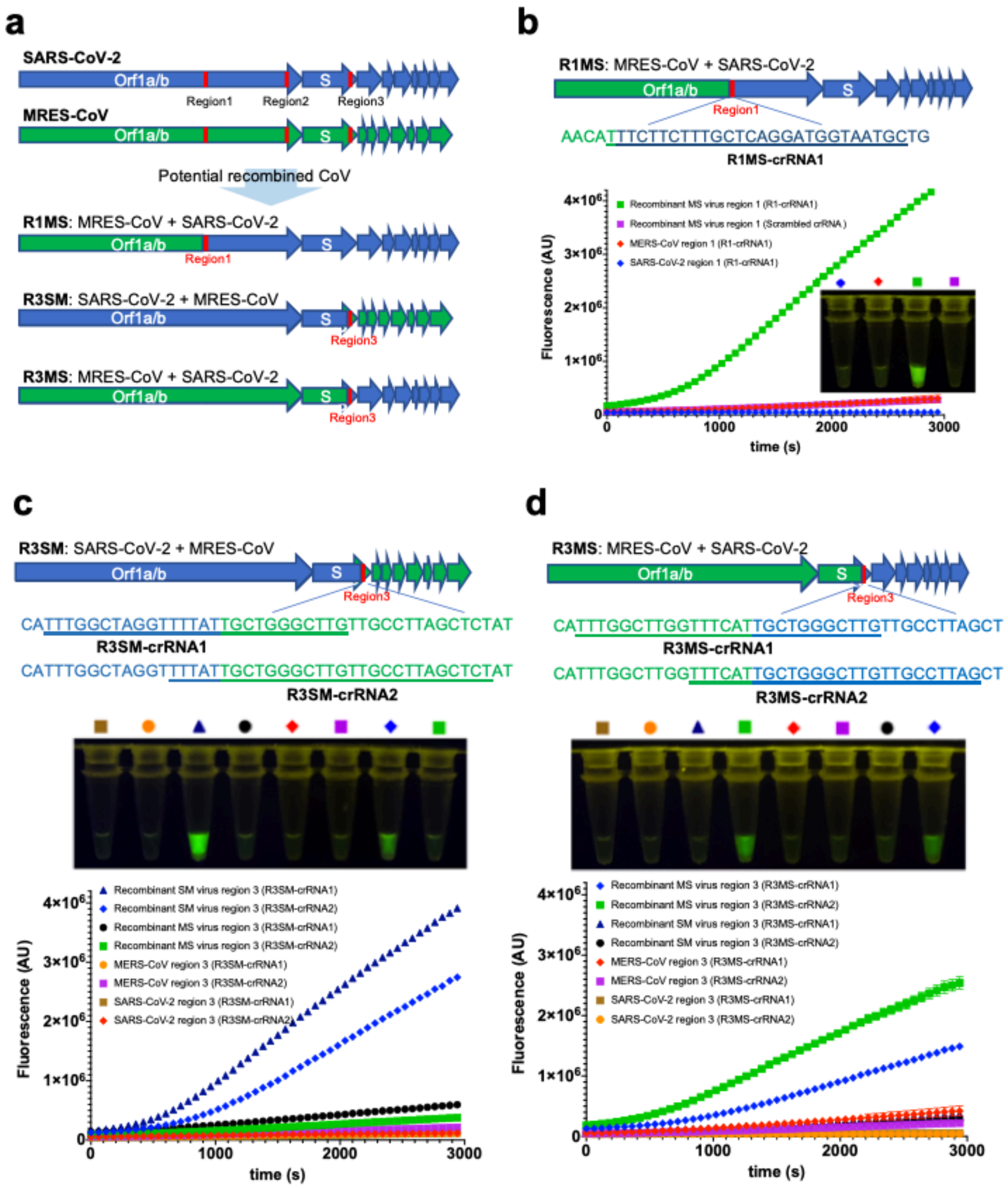

Figure S12. Detection of potential SARS-CoV-2 and MERS-CoV recombined virus. (**a**) Schematic diagram of SARS-CoV-2 and MERS-CoV recombined virus on three potential recombination regions[15]. Design and validate specific crRNAs for detection potential recombined virus on region 1 - R1MS virus (**b**), region 3 - R3SM virus (**c**) and R3MS virus (**d**).

Table S1

Table S1. Specific crRNAs targeting SARS-CoV-2 and MERS-CoV

crRNA name	crRNA target sequence (5' to 3')	Target Virus and Gene
SC2-crRNA1	TTTCTTGCTTTCGTGGTATTCTTGCTA	SARS-CoV-2, *E* gene
SC2-crRNA2	TTTCGTGGTATTCTTGCTAGTTACACT	SARS-CoV-2, *E* gene
SC2-crRNA3	TTTACAAGACTCACGTTAACAATATTG	SARS-CoV-2, *E* gene
SC2-crRNA4	TTTACTCTCGTGTTAAAAATCTGAATT	SARS-CoV-2, *E* gene
SC2-crRNA5	TTTTACAAGACTCACGTTAACAATATT	SARS-CoV-2, *E* gene
SC2-crRNA6	TTTGCTGCTGCTTGACAGATTGAACCA	SARS-CoV-2, *N* gene
SC2-crRNA7	TTTACCAGACATTTTGCTCTCAAGCTG	SARS-CoV-2, *N* gene
SC2-crRNA8	TTTCTTAGTGACAGTTTGGCCTTGTTG	SARS-CoV-2, *N* gene
SC2-crRNA9	TTTGTGACTTAAAAGGTAAGTATGTAC	SARS-CoV-2, *orf1a/b* gene
SC2-crRNA10	TTTGTACATACTTACCTTTTAAGTCAC	SARS-CoV-2, *orf1a/b* gene
MC-crRNA1	TTTGGACATATGGAAAACGAACTATGT	MERS-CoV, *E* gene
MC-crRNA2	TTTGTCCAAGAACGAATAGGGTTGTTC	MERS-CoV, *E* gene
MC-crRNA3	TTTACTATGAACAACCCTATTCGTTCT	MERS-CoV, *E* gene
MC-crRNA4	TTTCCATATGTCCAAAGAGAGACTAAT	MERS-CoV, *E* gene
MC-crRNA5	TTTCGTCAGCGCTGATTGCAGTTGCAA	MERS-CoV, *orf1a* gene
MC-crRNA6	TTTGCAACTGCAATCAGCGCTGACGAA	MERS-CoV, *orf1a* gene
R1MS-crRNA1	TTTCTTCTTTGCTCAGGATGGTAATGC	R1MS virus, *orf1a* gene
R3MS-crRNA1	TTTGGCTTGGTTTCATTGCTGGGCTTG	R3MS virus, *S* gene
R3MS-crRNA2	TTTCATTGCTGGGCTTGTTGCCTTAGC	R3MS virus, *S* gene
R3SM-crRNA1	TTTGGCTAGGTTTTATTGCTGGGCTTG	R3SM virus, *S* gene
R3SM-crRNA2	TTTATTGCTGGGCTTGTTGCCTTAGCT	R3SM virus, *S* gene

Note: The PAMs of LbCas12a were underlined. The constant region sequence of crRNA is UAAUUUCUACUAAGUGUAGAU.

Table S2

Table S2. Primers used in this study

Primer name	Sequence (5' to 3')
SC2-E-qPCR-F	ACAGGTACGTTAATAGTTAATAGCGT
SC2-E-qPCR-R	ATATTGCAGCAGTACGCACACA
SC2-E-probe	6-FAM-ACACTAGCCATCCTTACTGCGCTTCG-BHQ-1
SC2-E-RPA-F	CGGAAGAGACAGGTACGTTAATAGTTAATAGC
SC2-E-RPA-R	AGACCAGAAGATCAGGAACTCTAGAAGAAT
SC2-E-IVT-F	GATATCGGATCCCTAATACGACTCACTATAGGAGAATGTAC TCATTCGTTTCGGAAG
SC2-E-IVT-R	CGGGCCCTTTAAAAAAATTAGACCAGAAGATCAGGAACTC
MC-E-qPCR-F	GCAACGCGCGATTCAGTT
MC-E-qPCR-R	GCCTCTACACGGGACCCATA
MC-E-probe	6-FAM-CTCTTCACATAATCGCCCCGAGCTCG-BHQ1
MC-E-RPA-F	CTCGCTTATCGTTTAAGCAGCTCTGCGCTACTATG
MC-E-RPA-R	GTAGCCGTAAGGAAAGCCATACACACCAAGAGTG
MC-E-IVT-F	TAATACGACTCACTATAGGGAGATCTAGAAATAATTTTG
MC-E-IVT-R	GAATTCAAAAAAACACACATAATCTAG

Table S3

Table S3. qPCR detection of clinical samples

Clinical samples	Ct mean
CS1	33.80 ± 0.39
CS2	33.01 ± 0.12
CS3	Undetermined
CS4	36.82 ± 0.38
CS5	35.18 ± 0.02
CS6	Undetermined
CS7	32.77 ± 0.47
CS8	37.03 ± 0.21
CS9	Undetermined
CS10	Undetermined
CS11	Undetermined
CS12	37.95 ± 0.60
CS13	Undetermined
CS14	Undetermined
CS15	38.15 ± 0.50
CS16	35.17 ± 0.37
CS17	Undetermined
CS18	Undetermined
CS19	38.98 ± 1.63
CS20	33.87 ± 0.20
CS21	35.67 ± 0.67
CS22	34.22 ± 0.18
CS23	Undetermined
CS24	Undetermined
NC	Undetermined
PC	20.61 ± 0.30
